# Data-driven analysis of electrochemical impedance spectroscopy using the Loewner framework

**DOI:** 10.1016/j.isci.2025.111987

**Published:** 2025-02-10

**Authors:** Bansidhar Patel, Antonio Sorrentino, Tanja Vidakovic-Koch

**Affiliations:** 1 Max Planck Institute for Dynamics of Complex Technical Systems, Electrochemical Energy Conversion, Sandtorstrasse 1, D-39106 Magdeburg, Germany

**Keywords:** Electrochemistry, Mathematical method in physics

## Abstract

Optimizing electrochemical devices requires an understanding of the complex interplay between mass transport and electrokinetics at the electrode-electrolyte interface. Electrochemical impedance spectroscopy (EIS) is a powerful tool for probing these processes, with analysis typically performed using equivalent circuit models (ECMs). However, selecting the appropriate ECM is challenging, as different models can yield deceptively similar spectra, complicating the accurate representation of the underlying physics. This work presents a data-driven approach for extracting the distribution of relaxation times (DRTs) through the Loewner framework (LF), facilitating the identification of the most suitable ECM for a given EIS dataset. The method is validated on different variants of Randles ECMs, which are commonly used to describe electrochemical interfaces. Its robustness to noisy datasets, as well as its advantages over similar methods that employ inversion algorithms, are also discussed.

## Highlights

• Loewner framework (LF) advances analysis of electrochemical impedance spectroscopy
• LF provides unique distribution of relaxation times (DRTs)
• LF-derived DRTs distinguish between different equivalent circuit models (ECMs)
• Model discrimination validated by analyzing synthetic and experimental data

## Graphical abstract

**Figure:**
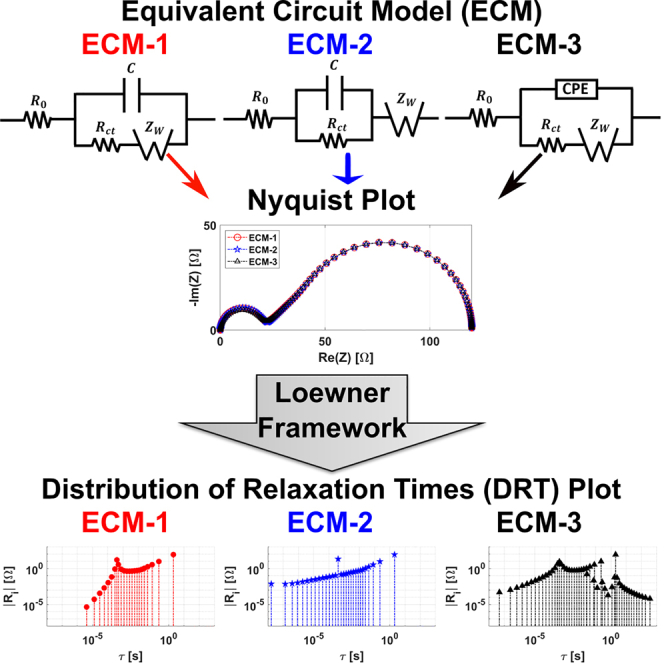

## Introduction

Electrochemical systems such as fuel cells, batteries, and electrolyzers are crucial for addressing global challenges like clean energy and sustainable fuels. However, issues with durability and reliability hinder their widespread adoption. Optimizing performance requires a deep understanding of the phenomena at the electrolyte-catalytic electrode interface, which governs efficiency. The complex interactions of transport and electrokinetic processes complicate the identification of performance-limiting factors. In this context, electrochemical impedance spectroscopy (EIS) stands out as one of the most useful experimental techniques for characterizing electrochemical systems due to its ability to measure separately the losses related to processes with different time constants. EIS provides valuable insights into the underlying electrochemical phenomena, such as charge transfer, mass and charge transport, different adsorption processes, and other electrochemical properties at the electrode-electrolyte interface.^1^^,^^2^^,^^3^^,^^4^^,^^5^

The EIS spectra are mostly analyzed through the fitting of so-called equivalent circuit models (ECMs).^1^^,^^3^^,^^6^ These are composed of lumped and distributed circuit elements, each resembling a particular physical process, that are placed in series and/or parallel to represent the dynamic electric response of an electrochemical device. They are, in some instances, based on physics based models offering the advantages of being very versatile, intuitive, and requiring low computational power. For these reasons, they are widely applied in algorithms for identifying faulty states and monitoring the state of health.

A meaningful interpretation of EIS data relies on selecting the most suitable ECM that reflects the specific electrochemical system under investigation. This is not an easy task, as different ECMs could apparently reproduce a certain EIS spectra with the same accuracy. This issue extends beyond EC modeling to other impedance analysis approaches, such as mechanistic-based modeling and the distribution of relaxation times (DRTs) analysis using inversion-based algorithms. Studies have shown that different mechanistic models can yield identical EIS responses.^7^^,^^8^ Regarding DRT, it has been demonstrated that multiple distributions can be derived and associated with a specific impedance spectrum, as the reconstructed impedances present often a quite good match to the data.^9^^,^^10^ This occurs because transforming impedance into a DRT is an ill-posed inverse problem. As a result, choosing the wrong model can lead to unreliable parameter estimation and misleading conclusions in general.^4^^,^^5^ Extracting accurate information from EIS spectra still requires a solid understanding of the underlying electrochemical phenomena and the ability to utilize appropriate ECMs for data fitting. Methods that enable model discrimination would be advantageous in improving these aspects.

In this publication, we report on such a method, known as the Loewner framework (LF). The LF is a data-driven approach developed by Prof. Antoulas at Rice University,^11^^,^^12^^,^^13^ and has been already applied to various examples in both academia and industry.^14^^,^^15^^,^^16^^,^^17^ In collaboration with his research group, we introduced the LF to the analysis of electrochemical systems, specifically for extracting DRT in discrete form from the LF’s linear state-space model.^18^^,^^19^^,^^20^^,^^21^ As demonstrated in our previous works,^20^^,^^21^ this algorithm computes a unique DRT for a given dataset without requiring arbitrary meta-parameters. It outperforms existing DRT extraction methods in terms of computational efficiency, accuracy, adaptability, user-friendliness, and versatility. The algorithm has been successfully employed to analyze both synthetic EIS spectra derived from common ECMs, as well as real experimental systems like as polymer electrolyte fuel cells^18^^,^^19^ and ferrocyanide oxidation reactions.^21^ Furthermore, our algorithm has also been adopted by others, as seen in studies by Danzer et al.^22^ and Rüther et al.^23^

To demonstrate the LF’s ability for model discrimination, both synthetic and real EIS data have been used. The synthetic EIS data were generated using four different ECMs. These ECMs are widely used to analyze impedances originating from charge transfer diffusion-coupled processes. Interestingly, these different models can result in the same EIS response, although they represent different physics of the electrochemical process. Differentiating between these model representations has always been a challenge, despite advancements in various impedance analysis approaches. This issue has been highlighted in recent publications by Orazem and Ulgut^24^ as well as in a publication by Franceschetti et al.^25^ from over three decades ago. Therefore, these models provide an excellent basis to demonstrate the LF’s ability for model discrimination. The detailed description of these models is provided in the next section. In addition to synthetic data, experimental data on the ferrocyanide reaction on the rotating disk electrode (RDE) have been used. These were selected because they perfectly match the assumptions of ECMs.

This paper is structured as follows: the methods section introduces the study cases and outlines the LF procedure for extracting DRTs from EIS spectra. In the results and discussion section, we first analyze the discrete DRTs of distributed elements, such as constant phase element (CPE) and Warburg, found in the investigated ECMs, and compare them to analytical derivations from previous literature. We then examine the DRTs of Randles ECMs, highlighting the qualitative differences that distinguish them, and propose criteria for model discrimination. Next, we assess the impact of noise on the DRTs to evaluate the robustness of our discriminative analysis. Subsequently, we examine experimental EIS spectra from a ferrocyanide reactive system to identify the corresponding ECM based on our proposed criteria. This approach tests the procedure on a real case where the processes underlying its EIS spectra are well known. The analysis is then repeated using a popular DRT algorithm based on Tikhonov regularization to compare its performance in model discrimination with the LF approach. Finally, we summarize the conclusions of this work.

### Methods

#### Study cases

The model discrimination is especially challenging in the case of electrochemical interfaces, where strong coupling between charge transfer and diffusion phenomena occurs.^24^^,^^26^ In these instances, among other models,^2^^,^^27^ various types of so-called Randles ECMs have been employed to model apparently identical EIS spectra, the patterns of which are observed in many systems.^2^^,^^28^^,^^29^^,^^30^^,^^31^^,^^32^ The most commonly used variants of Randles ECMs are illustrated in Table 1. ECM-1 is the classic Randles circuit named after its creator, who introduced it in a seminal contribution in 1947.^33^ This circuit comprises a resistor ($R_{0}$) representing the ohmic resistance in series with a parallel combination of a capacitor (*C*), embodying the double-layer capacitance, and a resistor ($R_{ct}$) associated with the charge transfer resistance. Additionally, a Warburg element ($Z_{W}$) is also placed in parallel to the capacitance to account for the effect of the diffusion of reactive species. The definition of Warburg element varies based on the diffusion type: semi-infinite linear diffusion, typically assumed in battery contexts,^34^ or finite-length diffusion (FLW), common when the diffusion layer’s thickness is well-defined (e.g., ferro/ferricyanide oxidation/reduction on RDEs^27^).^2^^,^^5^^,^^9^ Essentially, the Randles circuit model is based on the fact that diffusion and the kinetics of the reaction are intimately linked, as both strongly depend on the concentration at the surface of the electrode.^2^^,^^24^^,^^28^

**Table 1:** Variations of Randles equivalent circuit models used to describe electrochemical interfaces and their corresponding impedance expressions

	Equivalent circuit models	Impedance expressions
ECM-1		$Z_{ECM-1}=R_{0}+\frac{R_{ct}+Z_{W}}{1+j\omega C\left(R_{ct}+Z_{W}\right)}$
ECM-2		$Z_{ECM-2}=R_{0}+\frac{R_{ct}}{1+j\omega CR_{ct}}+Z_{W}$
ECM-3		$Z_{ECM-3}=R_{0}+\frac{R_{ct}+Z_{W}}{1+{\left(j\omega \right)}^{\emptyset }Q\left(R_{ct}+Z_{W}\right)}$
ECM-4		$Z_{ECM-4}=R_{0}+\frac{R_{ct}}{1+{\left(j\omega \right)}^{\emptyset }QR_{ct}}+Z_{W}$

The ECM-2 represents a departure from the original Randles model, where the Warburg element is positioned in series with the resistor-capacitor (RC) circuit unit. This variant was first introduced to study lithium-ion batteries by Levi and Aurbach,^35^^,^^36^ and since then, it has been extensively used in this field of research.^26^^,^^29^^,^^30^^,^^37^ Additionally, ECM-2 has been employed in impedance modeling of solid oxide fuel cells and sensors.^38^^,^^39^^,^^40^^,^^41^ Based on some authors its use is deemed appropriate when the supporting electrolyte is absent, meaning that the same chemical species serves as both the sole charge carrier in the migration process and the reactant in the electrochemical reaction simultaneously.^4^^,^^25^ This matches for example the physics of lithium-ion batteries.^4^ However, this opinion was recently challenged by Orazem and Ulgut^24^ who discussed the use of ECM-1 and ECM-2 in the context of batteries. Delving into the fundamental principles underlying the formulation of the Randles model, they stress the strong correlation between electrode reaction kinetics and the transport mechanisms facilitating the arrival of electroactive species at the electrode surface. They conclude that placing the Warburg element in series with all other elements (as in ECM-2) is only viable when the product of transport resistance and capacitance is sufficiently low, a condition based on these authors typically unmet in high-surface-area electrodes of batteries.

As the charging process can significantly deviate from that of a pure capacitor, a CPE is used in ECMs, as illustrated in the Table 1 for the ECM-3 and ECM-4. The reasons for deviation from the ideal case are generally attributed to surface roughness and electrolyte resistance dispersion. However, these two hypotheses are also under debate, as it is also claimed that adsorption of ionic species, often impurities, is mostly responsible for the CPE-type dispersion in EIS spectra (so called Frumkin and Melik-Gaykazyan impedance^35^^,^^36^^,^^42^), and that the roughness would even mitigate such effect in some cases. For instance, such phenomena have been observed in rotating disk planar electrodes.^42^ The deviation from the ideal capacitor is quantified by the exponent ∅=1 whose the value varies between 0 and 1. The closer its value is to 0, the more the behavior is non ideal. The introduction of a CPE can significantly enhance the model’s ability to fit experimental data, particularly in the high-frequency range, as validated across various references.^4^^,^^31^^,^^32^ However, the pure capacitance is sufficient to reproduce the data with good agreement in many cases.

#### LF-based algorithm for DRT extraction

This section describes the LF-based algorithm for extracting the DRT without delving into technical details. For a thorough mathematical introduction to the LF, the references,^11^^,^^12^^,^^13^^,^^14^^,^^15^ written by Antoulas and collaborators on the topic, can be consulted. The description of the algorithm to compute the DRT is detailed in references,^18^^,^^20^^,^^21^ as well as in the STAR Methods section. Additionally, the related MATLAB code is available online in its latest version.^43^

**Figure 1:**
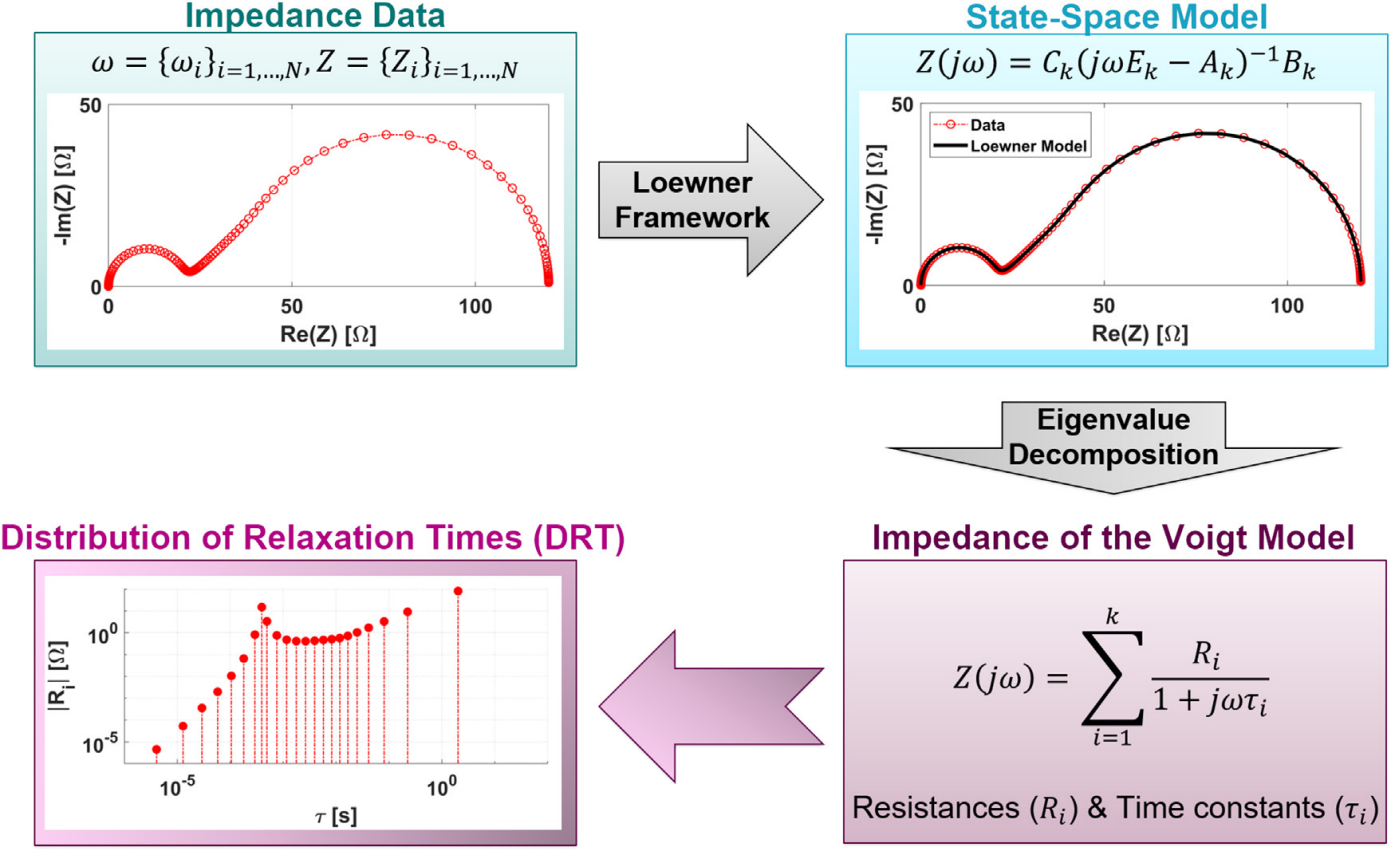
Schematic of the Loewner framework-based algorithm for calculating the DRT

As shown in Figure 1, given a set of EIS data points, the LF generates a linear state-space model that interpolates them. An eigenvalue decomposition of the state-space matrix of the model allows the impedance to be expressed as a sum of first-order transfer functions (see the equation in the box in Figure 1). This is analogous to the impedance response of a so-called Voigt model, which consists of a circuit with multiple RC circuits connected in series. By plotting the resistances ($R_{i}$) against their corresponding time constants ($\tau_{i}$), a discrete DRT is obtained, typically reported on a semi-log scale. However, for our purposes, we present the data on a log-log scale throughout the paper, which enhances the DRT graphs for small τ-values.

This LF-based DRT extraction method offers several advantages over traditional inverse algorithms, such as those based on Tikhonov regularization. Firstly, it does not rely on iterative optimization techniques, which can be computationally expensive. Additionally, it eliminates the need for setting meta-parameters, thereby providing a direct and unique DRT for a given dataset without the need to adjust the algorithm.

It should be noted that many researchers have explored the possibility of representing EIS data with a generalized Voigt model. For instance, Agarwal and Orazem employed this method to analyze EIS data, verify compliance with the Kramers-Kronig relation, and identify the number of underlying processes in their measurements.^44^^,^^45^^,^^46^ Other groups employed it to describe distributed elements, such as Warburg impedance.^30^^,^^47^ However, these procedures rely on iterative algorithms and require repeated fitting with an increasing number of first-order transfer functions to achieve an acceptable approximation. In contrast, the LF algorithm provides the same information in a single run.

## Results and discussion

### Analysis of the distributed ECM components

The introduction of CPE and Warburg elements in ECMs is essential for the analysis of electrochemical interfaces, as they accurately describe how the electrical response is distributed over time and space. These distributions are primarily due to the diffusion of electroactive species and inhomogeneities in the electrode surface. Unlike ideal circuit elements, the mathematical expressions for CPE and Warburg elements are characterized by irrational functions. This means that they can only be perfectly represented by an infinite sum of first-order transfer functions, or, in other words, by a Voigt circuit with an infinite number of RC elements.^20^^,^^21^^,^^30^^,^^47^ Therefore, the finite sum obtained through the LF method provides an approximation of the ideal solution.

**Figure 2:**
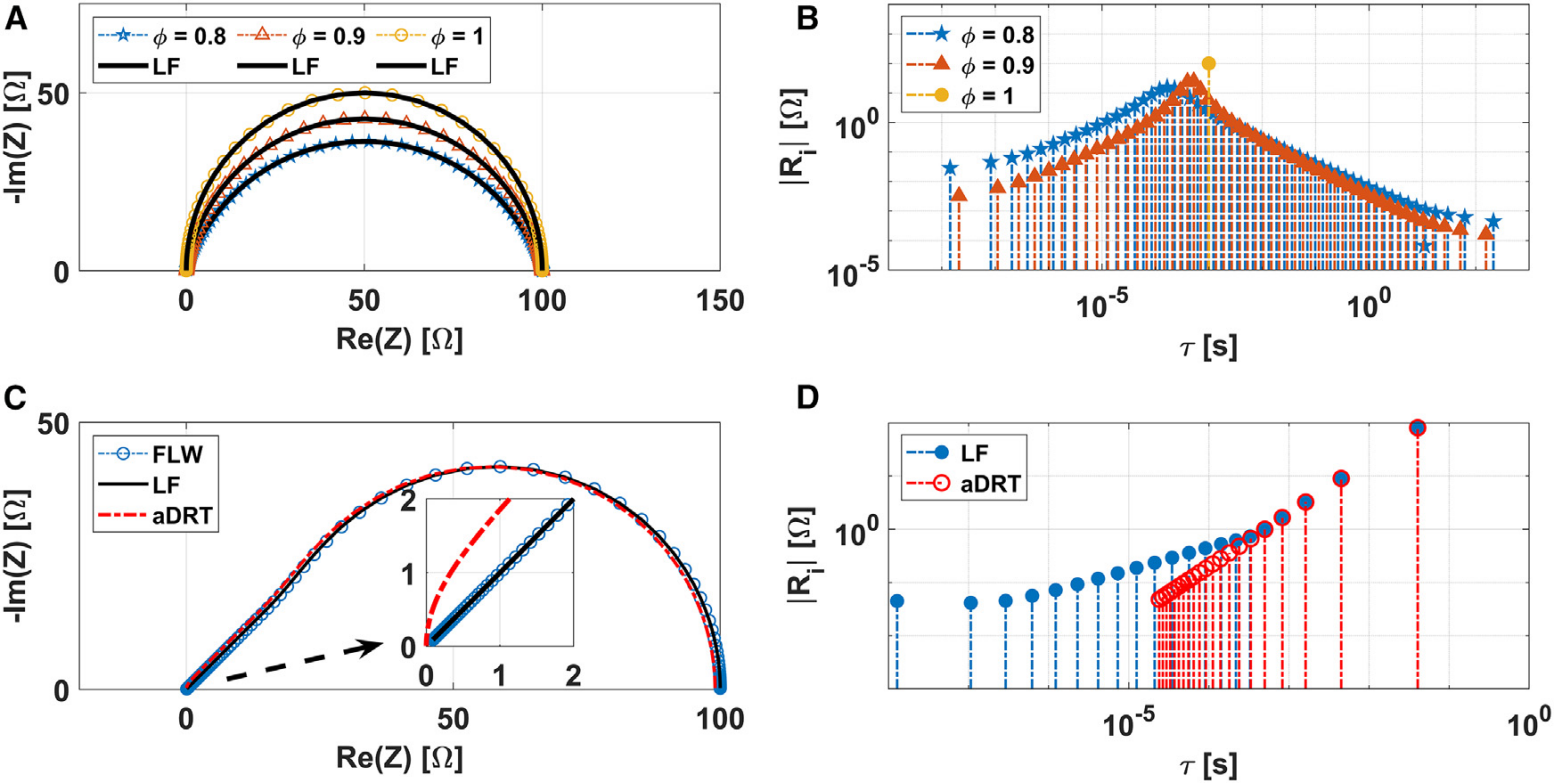
LF analysis of individual elements of the ECMs (A) Nyquist plot representation of the R-CPE EC. (B) Corresponding DRTs for the R-CPE EC, highlighting the influence of the parameter ∅. (C) Nyquist plot representation of the FLW. (D) Corresponding DRTs for the FLW.

Before analyzing the combined effects of CPE and Warburg elements in Randles ECMs, we first test the ability of the LF-based algorithm to extract the DRT for each element individually. For the Warburg behavior, we also compare our DRT results with analytical approximations from other studies.^9^^,^^10^^,^^30^^,^^47^^,^^48^ Figure 2 presents the Nyquist plots (A) and DRTs (B) for an R-CPE circuit determined through the LF-based method. The effect of the parameter ∅, which quantifies the deviation from an ideal capacitor is analyzed (refer to the Equation S1 in the supplemental information). As anticipated, reducing the value of ∅ causes the Nyquist plot spectra to transition from a perfect semicircle (∅ = 1), similar to that of an RC circuit, to a more depressed shape (∅<1). Similarly, the computed DRTs shift from being represented by a single impulse function (the expected DRT for an RC circuit) to a Gaussian-type distribution of impulses, shown here in log-log scale. The sum of the resistance peaks $R_{i}$ represented by all the impulse functions is equivalent to the resistance of the R-CPE circuit ($R_{CPE}=\sum R_{i}$). The difference between the DRTs of RC and R-CPE circuits allows us to distinguish between simple and complex capacitive behaviors. This distinction is not possible with many DRT algorithms that use Gaussians as basis functions, which provide continuous distributions.

Figures 2C and 2D compares the DRTs obtained by applying the LF-based algorithm to EIS spectra containing FLW with analytical approximations (aDRT) proposed in the literature (also referred to as Equation S4 in the supplemental information) to describe the same pattern.^9^^,^^49^ The LF-based DRT displays a prominent impulse function at the characteristic time constant of the FLW, followed by smaller, decreasing impulse functions at lower time constants. This trend matches perfectly with the analytical predictions for the first five impulse functions. However, discrepancies arise in the distribution at high frequencies. Interestingly, the LF method accurately reproduces the EIS spectra across the entire frequency range, as demonstrated in Figure 2C. In contrast, the analytical approximation struggles to simulate the FLW accurately at high frequencies. The accuracy of both methods is compared in Figure S1 of the supplemental information, which shows the residuals between the generated synthetic Nyquist plot and those obtained from the two methods.

Regarding the analytical approximation of the DRT, to achieve accurate simulation, an infinite number of elements (k→∞), would need to be accounted in the equation (see the Equation S4 in the supplemental information) of the related model. As observed, the LF achieves a better accuracy by optimally positioning its time constants (or poles), resulting in fewer elements required for rational interpolation. Further investigation is needed to explain these discrepancies, but this is beyond the scope of this article.

It is crucial to recognize that all inversion methods for calculating DRT face challenges in accurately reproducing the distribution due to the asymmetric nature of the FLW impedance.^10^ Please note that all examined ECs demonstrate unique DRTs, which facilitate a deeper understanding of the underlying physics. For additional examples, refer to our prior work.^21^ Therefore, it is showed that our approach effectively computes both rational and irrational transfer functions, enabling the distinction between lumped and distributed behaviors through DRT analysis. The implications of this capability are explored further in the next section.

### Identification of the Randles ECMs variants in noise-free conditions

**Figure 3:**
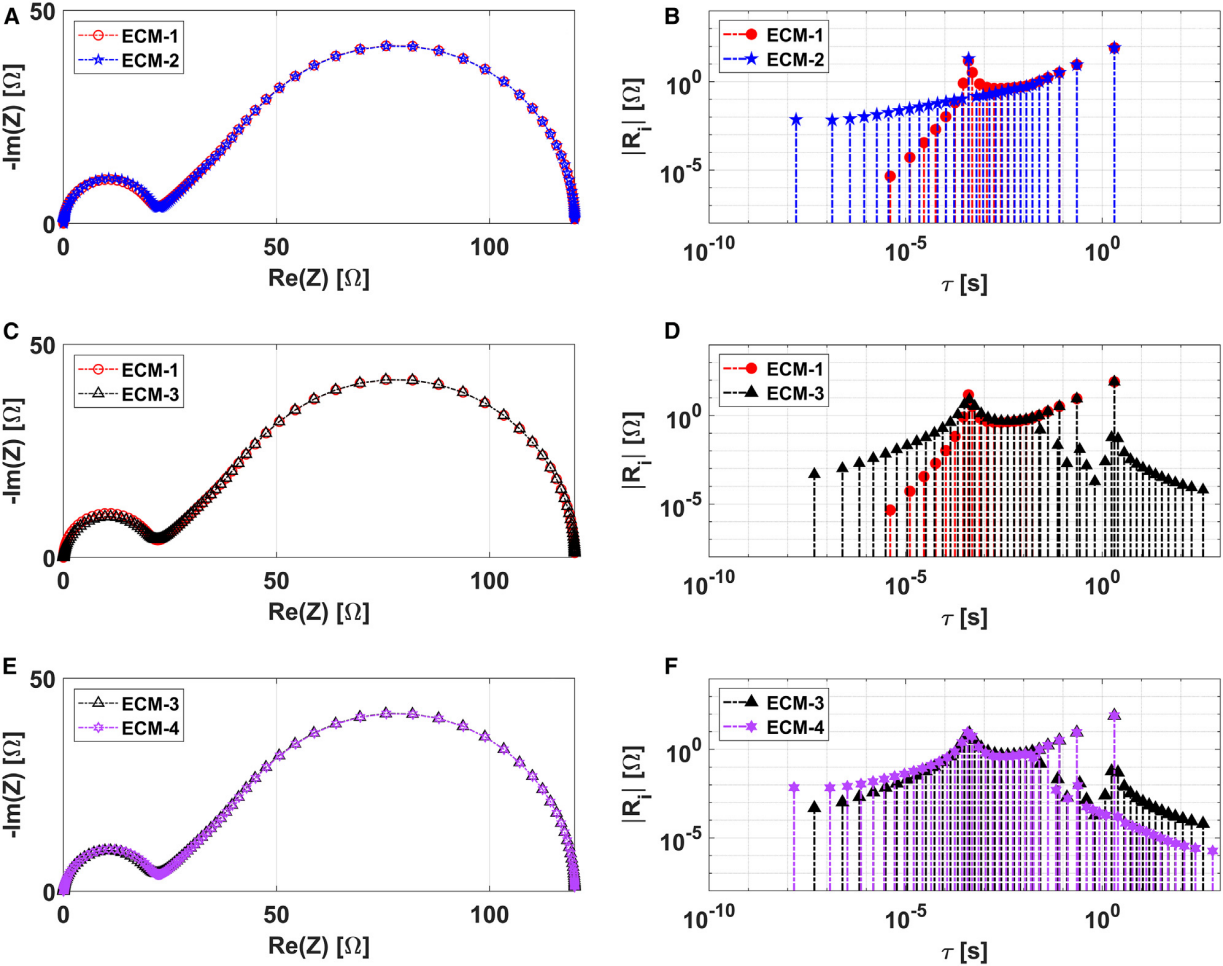
Analysis of simulated EIS data corresponding to different ECMs (A, C, and E) Nyquist plot representations. (B, D, and F) Corresponding DRT plots.

In Figure 3, Nyquist plots and DRTs related to the Randles ECMs depicted in Table 1 are compared. The same parameter values have been used for different elements in the ECMs (see Table S1 in the supplemental information for more details). For simplicity, the solution resistance ($R_{0}$), which is additive in all cases, has been neglected. The EIS data points are uniformly determined across a frequency range spanning from 10^−3^ to 10^6^, with 15 points per decade (ppd), using a logarithmic scale.

While the Nyquist plots are nearly identical, the DRTs computed using the LF-based algorithm exhibit some distinct characteristics. For instance, both ECM-1 and ECM-2 (Figure 3B) display patterns related to charge transfer resistance ($R_{ct}$) localized at a time constant range of 10^−4^ to 10^−3^ s, and another for the Warburg element (FLW) characterized by the descending pulse functions extending from 5 to 10^−8^ s. However, the distribution associated with $R_{ct}$ presents different features between the two cases. In the DRT related to ECM-1, it shows a distributed behavior similar to the R-CPE. In contrast, for ECM-2, $R_{ct}$ appears as a single pulse function, suggesting a resemblance to an RC element. Then, it is remarkable that this difference allows for a clear distinction between the two, which imply a different interaction between the charge transfer and the reactant diffusion. This was also verified on the same ECM configurations contemplating semi-infinite linear Warburg diffusion (see Figures S2B and S2D in the supplemental information).

It is intriguing that the DRT for ECM-1 (Figure 3B) displays a behavior characteristic of a CPE, even though the model includes a pure capacitor as component. For clarity, we describe this behavior as “apparent CPE” rather than actual CPE. From a mathematical perspective, the reason is clear: the transfer function related to this circuit is an irrational function (see related Equation in the Table 1). Therefore, even though an ideal capacitor is in parallel with an FLW and $R_{ct}$ element, it resembles a distribution of impulse functions similar to the one of a CPE element. Moreover, the observed pattern is also in line with the physical understanding of reactive diffusive electrochemical systems. For instance, in the case of a planar electrode, the faradaic processes occurring in parallel with the double layer charging at the interface is directly influenced by the diffusion of the electroactive species.^27^ For this reason, the distributed time behavior of the latter influences the response of the former process. Further evidence about the apparent CPE is discussed in the section concerning the analysis of experimental impedance spectra.

The behavior of the apparent CPE is also obtained through simulation of the analytical DRT approximation of the ECM-1 model derived by Montella.^49^ A comparison between these two DRTs is shown in Figure S3 of the supplemental information. A perfect qualitative match is observed, further confirming the distribution that arises from the strong interplay between the kinetics and diffusion elements.

The presence of apparent CPE-type behavior in the DRT plot could potentially lead to incorrect interpretations, as it could be misunderstood as an actual CPE. To verify this, we compared the DRTs related to ECM-1 and ECM-3 in Figure 3C. The two models present the same circuit configuration aside from the fact that the former contains a pure capacitance, while the latter a CPE. To ensure a fair comparison, we set the CPE exponent to ∅=0.95, and the CPE parameter Q was adjusted according to the formula $Q={\left(RC\right)}^{\emptyset }/R$.

As shown (Figure 3D), both models display a similar CPE-type distribution of pulses in the middle frequency region. However, despite these similarities, significant differences exist. Specifically, the ECM-3 (black diagram) features a wider distribution compared to that of ECM-1 at very low time constants. This is attributed to the distributed nature of the actual CPE. This widening effect is more pronounced at lower values of the CPE exponent ∅, as depicted in Figure S4.

Moreover, a different trend in the distribution is observed exclusively in the case of ECM-3 at higher time constants (10^−2^-10^3^ s). This occurs because the CPE impulse functions are distributed across multiple decades of time constants and interact with the Warburg element. This is clearly illustrated in the Figure S5, which displays the DRTs of ECM-3 along with those of the individual CPE and Warburg elements. These distinctions could enable the discrimination between apparent CPE and actual CPE and, consequently, between ECM-1 and ECM-3.

However, distinguishing between ECM-3 and ECM-4 is nearly impossible, as both conditions produce identical spectra in the Nyquist plot (Figure 3E) and in the DRT plot (Figure 3F). In this case, the analysis of the DRTs is also insufficient for discriminating the position of the Warburg element and, ultimately, the underlying physics of the analyzed system.

### Effect of noise on the identification of Randles ECMs

Impedance spectra obtained from real systems invariably contain measurement errors or noise, stemming either from the testing equipment or the experiment itself.^50^ To assess the robustness of our approach, we introduced normally distributed noise into the synthetic data, employing a method akin to that described in literature.^51^ This adjustment was made in accordance with the equation as follows:

$$Z_{\text{noise}}=Z_{\text{real},\text{noise}\;}+jZ_{\text{imag},\text{noise}\;}$$

where

$$\;Z_{\text{real},\text{noise}}=Z_{\text{real}}+N_{1}\left(0,\sigma^{2}\right),\text{and}\;Z_{\text{imag},\text{noise}}=Z_{\text{imag}\;}+N_{2}\left(0,\sigma^{2}\right)$$

The standard deviations for the real and imaginary parts of the impedance were chosen to be equal, with a value of σ=0.002|Z| as recommended in a referenced paper,^51^ aligning with experimental observations.

**Figure 4:**
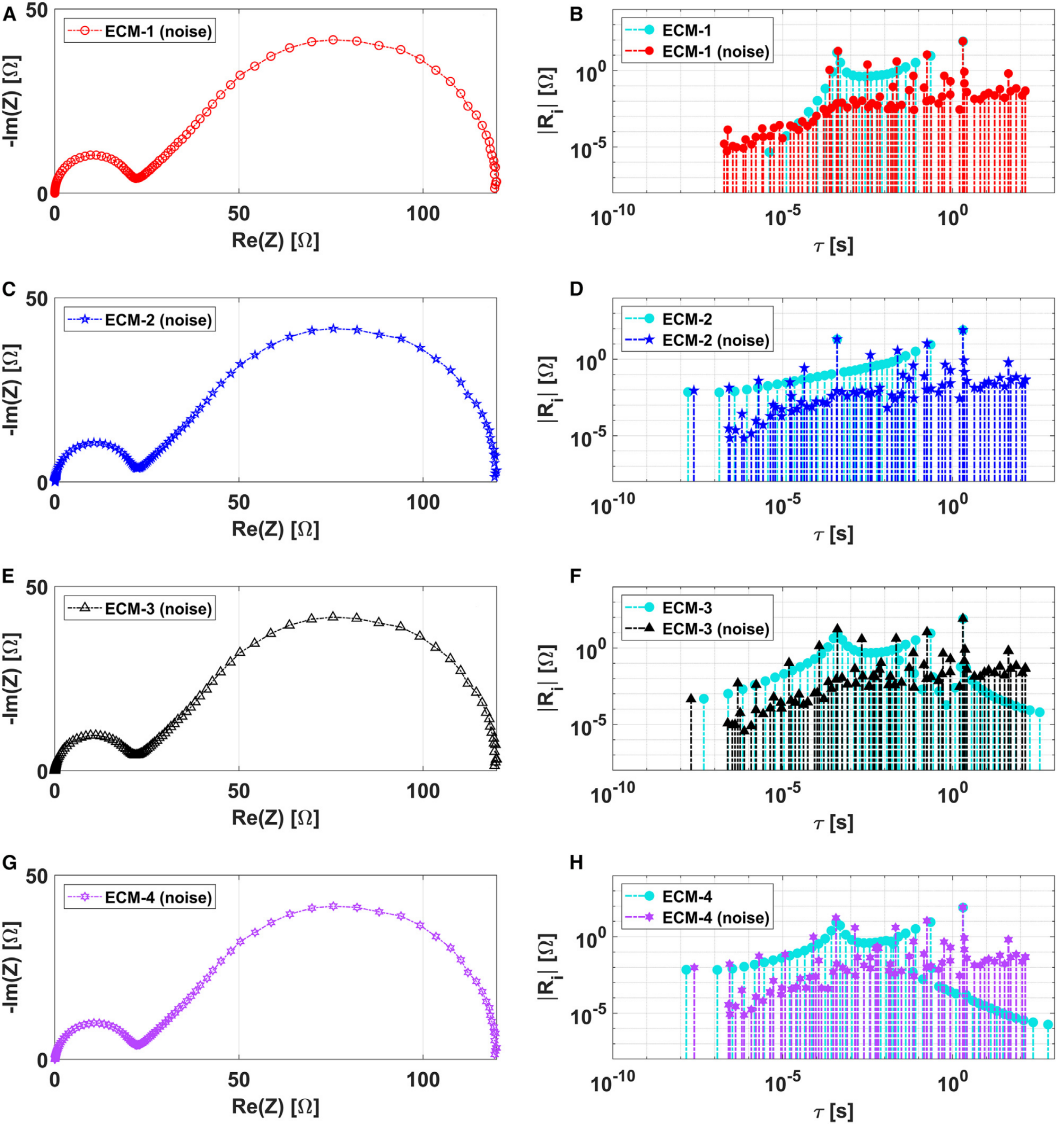
Analysis of simulated noisy EIS data corresponding to different ECMs (A, C, E, and G) Nyquist plot representations. (B, D, F, and H) Corresponding DRT plots.

Figure 4 displays the corrupted EIS spectra alongside the corresponding DRTs for all four Randles ECMs. The noise-free DRTs, depicted in cyan, are also plotted to facilitate comparison. Noise presence markedly affects the obtained DRTs, leading to a reduction in the number of significant pulse functions in the noisy DRT compared to its noise-free counterpart. As shown in the Figure S6, they reduce further by increasing the level of noise.

This phenomenon is consistent with our previous works and aligns with findings reported in the existing literature. Agarwal et al.^44^ observed a decrease in the number of elements required for a Voigt model to fit the EIS data of an EC model as noise levels increased. Experimental work by Lagonotte et al.^52^ on a classical ferri-ferrocyanide electrochemical system under equilibrium conditions demonstrated that a Voigt model with only a few elements sufficiently fits the experimental EIS data. Montella’s^49^ recent study offers a mathematical rationale for the adequacy of a Voigt circuit, comprising only a few RC elements, in accurately representing the impedance data of such systems in practical scenarios.

It is noticeable that the DRT plots (Figure 4) reveal additional peaks of lower magnitude that are absent in the noise-free scenario. These are also attributed to the presence of noise. The main reason of this additional feature is that the LF is a direct interpolation method and, therefore, is prone to overfitting. When noise is present, the algorithm calculates extra poles in the rational polynomial to match the noise-affected data, resulting in peaks that are not related to physical processes. However, even with a high level of artificially inserted noise in the simulated data, these anomalies remain minor.

Yet, noise makes the discrimination of the different models more difficult, although it remains possible in some cases. For instance, the DRT related to ECM-1 can still be distinguished from those of the other ECMs, as the broadening of the distribution at lower time constants, which differentiates the latter, remains preserved. On the other hand, the DRT of ECM-2 tends to become indistinguishable from those of ECM-3 and ECM-4 as the level of noise increases (as shown in Figure S6 of the supplemental information). Nonetheless, the distinct peak associated with the RC lumped element in ECM-2 remains identifiable, even at the highest noise levels considered.

Therefore, it can be concluded that the arrangement of the Warburg element (whether parallel or series) within the faradaic process, and its associated physical meaning, can only be clearly determined when the observed capacitance approximates ideal behavior.

### Analysis of experimental EIS data

In this section, the proposed methodology is applied to identify the appropriate Randles model variant describing a redox ferrocyanide system directly from experimental data. The measurements were performed using a RDE setup, with detailed experimental procedures outlined in the STAR Methods section as well as in studies by Vidaković-Koch et al.^27^ and Panić et al.^53^

**Figure 5:**
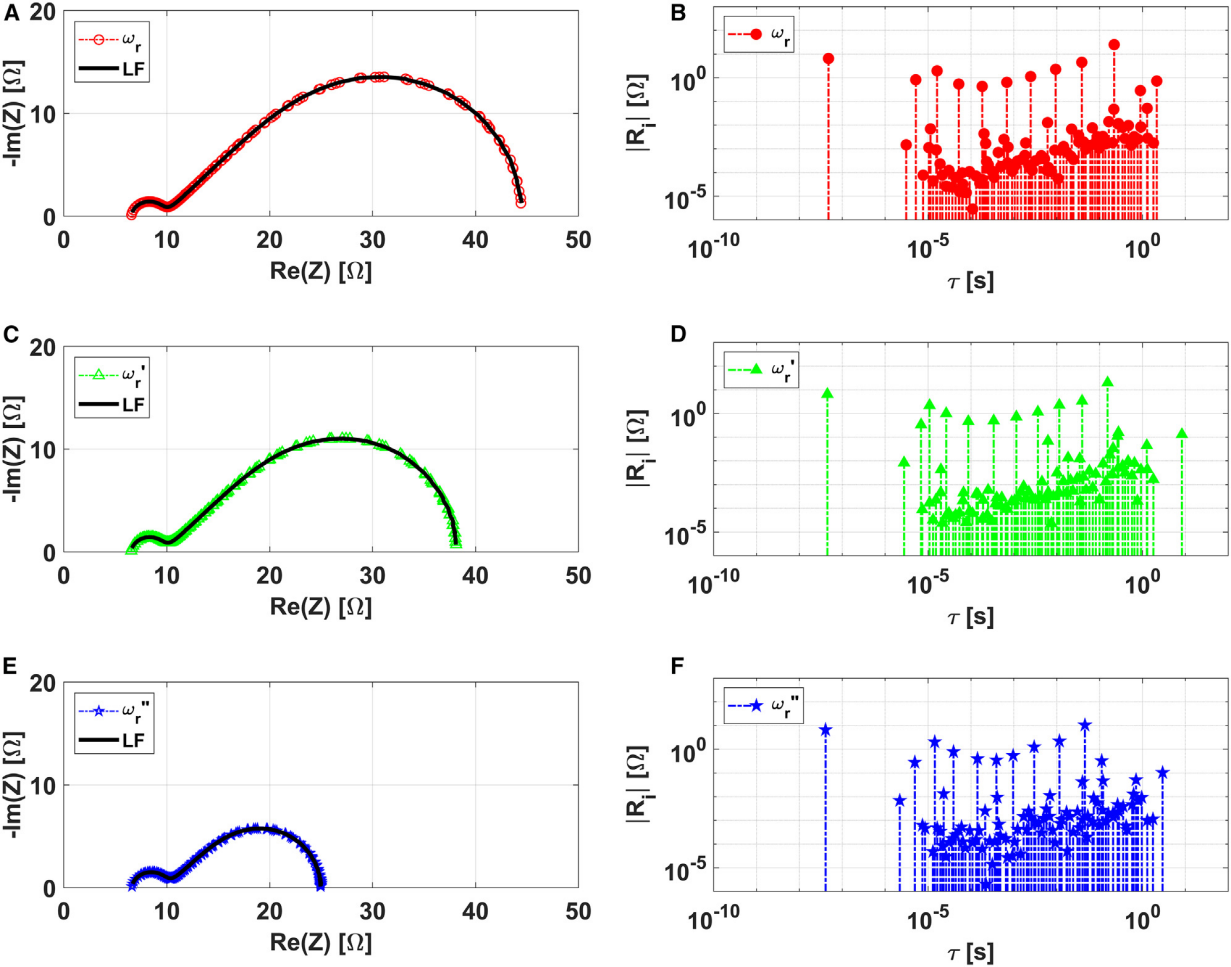
Analysis of experimental EIS data of ferrocyanide oxidation at different rotation rates ($\omega_{r}=600\;rpm$, ${\omega_{r}}^{\prime }=900\;rpm$, and ${\omega_{r}}^{\prime \prime }=3200\;rpm$) (A, C, and E) Nyquist plot representations. (B, D, and F) Corresponding DRT plots.

Figures 5A, 5C, and 5E display the EIS spectra collected at equilibrium conditions for three different rotation speeds, denoted as $\omega_{r}=600\;\text{rpm}$, ${\omega_{r}}^{\prime }=900\;\text{rpm}$, and ${\omega_{r}}^{\prime \prime }=3200\;\text{rpm}$. This experimental system largely satisfies requirements of models introduced in the previous section. Accordingly, the higher frequency arc in the Nyquist plot is attributed to kinetic processes, while the lower frequency arc to mass transport resistance.^27^^,^^53^Figures 5B, 5D, and 5F illustrate the corresponding DRT plot, which aligns with these observations.

It is remarkable that a prominent impulse function is observed at low time constants, which was not present in the DRTs previously analyzed. This is related to the ohmic resistance, as also suggested by the comparison of its value with the intercept of the Nyquist plot with the real axis at high frequencies. As discussed in our previous works,^20^^,^^21^ since the LF-based algorithm interpolates the data with a Voigt circuit, such an element is computed as an RC with extremely low time constants ($10^{-8}\;\text{s}$ in this case).

A careful examination of the DRTs indicates a similarity with that corresponding to the ECM-1. As seen in Figure 4, the distribution tail observed at lower time constants ($10^{-4}-10^{-8}\;s$) for all the other ECMs is absent in this case. This finding matches with the physical picture of the ferrocyanide oxidation system. In this context, the interfacial electrochemical reaction, facilitated by diffusion, typically sees the rate of the faradaic reaction being limited by the diffusion of electroactive reactants to the electrode’s surface.^2^^,^^27^^,^^33^ Consequently, diffusion and reaction kinetics are intimately connected, with the differential equations that describe diffusion being solved alongside the boundary condition determined by the reaction rates at the surface.^24^ Therefore, the ECM used to represent this system should include a diffusional resistance element (FLW) in series with the charge transfer resistance ($R_{ct}$), both in parallel with C^27^^,^^53^ or CPE,^24^^,^^32^ as indeed contemplated for the ECM-1. This is further corroborated by the observation that the chosen CPE parameter closely approximates a real double layer. This suggests that the experimental system behaves nearly as an ideal double layer, exhibiting apparent CPE behavior as previously introduced. Therefore, the use of the ECM-1 configuration for this system is well justified.

In summary, analyzing impedance spectra in systems featuring both diffusion and charge transfer requires careful attention due to the intricate interplay between these phenomena. This interaction can complicate the selection of the appropriate ECM and may result in the mistaken identification of CPE due to the asymmetric characteristics of distributed processes like Warburg effects. However, the proposed analysis facilitates the accurate determination of the true ECM components in case of capacitive behavior close to ideality.

### Comparison with inverse DRT extraction algorithms

In this section, the LF approach has been compared with a Bayesian-based inversion algorithm DRT tools.^54^^,^^55^^,^^56^^,^^57^ At first the regularization based DRT was calculated for individual elements of the ECMs, i.e., CPE (see Figures S7A and S7B in supplemental information) and FLW element (see Figures S7C and S7D in supplemental information). As can be seen the distribution for the CPE shows a characteristic single peak, resembling the trend of the impulse functions of the DRT extracted from the LF for the same element (refer to Figure 2). In contrast, the distribution for the Warburg element exhibits different descending Gaussian-type peaks. Although these peaks resemble the corresponding LF-based DRT, they lack the same monotonicity and smoothness along the time constants. This discrepancy likely arises from the use of Gaussian functions as the basis in the optimization algorithm. As noted in various publications,^10^^,^^58^ this assumption hinders the algorithm’s ability to reproduce the impulse function characteristic of such distributions, as well as those related to an RC element. We believe that the multiple peaks observed over a wide range of time constants when computing DRTs for distributed behaviors like the FLW by using the inversion algorithm can lead to misinterpretations of EIS spectra. In studies on fuel cells and batteries, where Warburg patterns may appear, each peak is often attributed to a different process.^59^^,^^60^^,^^61^ In reality, several peaks can correspond to the same process. This issue has also been highlighted by Dierickx et al.^62^

**Figure 6:**
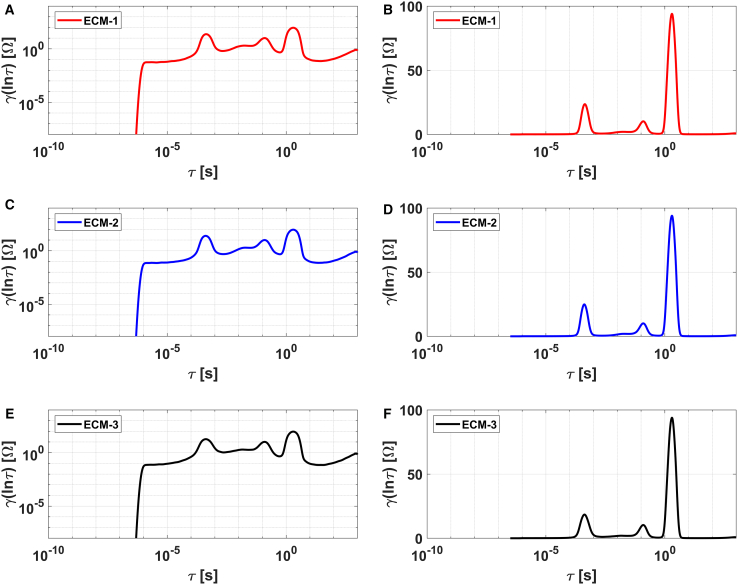
DRTs extracted using a Bayesian-based inversion algorithm through DRT tools for different variations of the Randles ECM (ECM-1, ECM-2, and ECM-3) (A, C, and E) DRT plots in log-logarithmic scale. (B, D, and F) DRT plots in semi-logarithmic scale.

Finally, the DRTs in semi-logarithmic scale for different Randles ECM models, computed using the inversion algorithm, exhibit multiple peaks that are difficult to associate with a single process (see Figures 6B, 6D, and 6F), which complicates their accurate interpretation. In contrast, the same distributions shown in log-log scale in Figures 6A, 6C, and 6E display identical quantitative and qualitative trends, making them indistinguishable from one another. Consequently, this method for extracting the DRT is not suitable for discriminating between ECMs, unlike the LF-based algorithm.

### Conclusion

This study examined the effectiveness of a data-driven approach using the LF for analyzing EIS data and discriminating between different models. As a case study, 4 Randles ECM variants have been selected. The analysis of the DRT obtained through the LF revealed distinct features for different ECMs, even when their Nyquist plots were nearly identical. Notably, the algorithm could differentiate between apparent CPE behavior arising from the coupling of diffusion and charge transfer, and actual CPE behavior due to the presence of a CPE in the ECM. The impact of noise on the extracted DRTs was also investigated. It was found out that while noise reduced the number of significant pulse functions, the main features of the DRTs remained distinguishable as long as the capacitive behavior was nearly ideal. The proposed methodology was applied to experimental EIS data from a ferrocyanide redox system, successfully identifying the correct ECM variant based on the DRT analysis. In other words, the identified ECM matches the physical picture of the investigated system. A comparison with an inverse algorithm widely used in the literature shows that the use of the LF highlights the pitfalls of interpreting DRT diagrams, particularly in the presence of distributed behaviors like the Warburg impedance, which can lead to misinterpretations of EIS spectra. In conclusion, the LF-based approach emerges as a beacon of accuracy, paving the way for precise ECM discrimination and analysis of electrochemical interfaces.

### Limitations of the study

This study primarily analyzes various Randles ECM variants, demonstrating the proposed method’s effectiveness in model discrimination. The experimental data examined here originate from a simple ferrocyanide redox system. However, the method’s applicability for discriminating ECMs related to more complex electrochemical systems, such as lithium-ion batteries, remains to be explored. While the findings highlight the method’s potential to enhance electrochemical analysis, its reliance on expert input for ECM selection limits its practicality. Developing an automated algorithm for independent model selection would significantly improve usability and expand its applicability across diverse electrochemical systems. Additionally, the method faces challenges in distinguishing models like ECM-3 and ECM-4, which involve multiple highly distributed processes such as CPE and FLW elements. Addressing these limitations with more advanced strategies could enhance the method’s versatility and validate its effectiveness across a broader range of electrochemical applications.

## Resource availability

### Lead contact

Further information and requests for resources should be directed to and will be fulfilled by the lead contact, Bansidhar Patel (patel@mpi-magdeburg.mpg.de).

### Materials availability

The study did not generate unique materials.

### Data and code availability

• All the data discussed in this paper are available on the Edmond repository platform. The link for the dataset is listed in the key resources table.
• All code used in this study to generate synthetic data and perform analysis is available on the Edmond platform and the GitHub repository. The links to the code are provided in the key resources table.
• Any additional information required to reanalyze the data reported in this paper is available from the lead contact upon request.

## Acknowledgments

All authors sincerely thank Dr. Vladimir Panic for providing the experimental data, which was collected during his postdoctoral research under the supervision of Prof. Kai Sundmacher at the Max Planck Institute for Dynamics of Complex Technical Systems in Magdeburg. B.P. is affiliated with the International Max Planck Research School for Advanced Methods in Process and Systems Engineering (IMPRS ProEng), Magdeburg, Germany.

## Author contributions

Conceptualization, B.P., A.S., and T.V.-K.; methodology, B.P.; investigation, B.P.; formal analysis, B.P.; software, B.P.; writing – original draft, B.P.; writing – review & editing, B.P., A.S., and T.V.-K.; funding acquisition, T.V.-K.; resources, T.V.-K.; supervision, A.S. and T.V.-K.

## Declaration of interests

The authors declare no competing interests.

## Declaration of generative AI and AI-assisted technologies

During the preparation of this work, the authors used ChatGPT 4 developed by Open AI in order to refine the language in certain parts of the manuscript. After using this tool, the authors reviewed and edited the content as needed and take full responsibility for the content of the publication.

## STAR★Methods

### Key resources table

**Table:** 

REAGENT or RESOURCE	SOURCE	IDENTIFIER
**Deposited data**		

Experimental data	This paper; Edmond	https://doi.org/10.17617/3.MMLZAK
Code for the analysis of experimental data	This paper; Edmond	https://doi.org/10.17617/3.MMLZAK
Code for the analysis and generation of simulated data	This paper; Edmond	https://doi.org/10.17617/3.MMLZAK

**Software and algorithms**		

MATLAB	MathWorks	https://www.mathworks.com
DRT tools	GitHub	https://github.com/ciuccislab/DRTtools
DRT-from-Loewner-framework	GitHub	https://github.com/projectsEECandDRI/DRT-from-Loewner-framework
ZPlot	Scribner Associates	http://www.scribner.com/general-electrochemistry-soft-ware.html

**Other**		

Electrochemical cell setup	Panić et al.^53^	https://doi.org/10.1021/jp201300a
Solartron 1287 potentiostat/galvanostat	Solartron	N/A
Solartron 1250 frequency response analyzer	Solartron	N/A

### Method details

#### Experimental details

The experiments were conducted in a standard three-electrode electrochemical cell, featuring a glassy carbon RDE as the working electrode (0.20 cm^2^), a platinum wire as the quasi-reference electrode, and a platinum mesh as the counter electrode. The electrolyte, purged with N_2_, was an equimolar solution of K₄[Fe(CN)₆] and K₃[Fe(CN)₆], each at a concentration of 20 mmol/dm³, containing 1.0 mol/dm³ KCl as the supporting electrolyte. The open-circuit potential readings consistently ranged within ±10 μV. Before each immersion into the solution, the glassy carbon disk was polished with 0.05 μm alumina and cleaned in an ultrasonic bath for a few seconds. Measurements were performed at 25.0°C using a Solartron 1287 potentiostat/galvanostat coupled with a Solartron 1250 frequency response analyzer. Periodic potential perturbations with an amplitude of 10 mV rms around the open-circuit (equilibrium) potential were applied as input, while separate harmonics, up to the third order, of the periodic output current signal were recorded. Technical details regarding the recording of harmonics can be found in the Help files of ZPlot software, Scribner Associates, Southern Pines, NC, 2007. (http://www.scribner.com/general-electrochemistry-soft-ware.html). All measurements were conducted within the frequency range of 65 kHz to 1 mHz. To examine the influence of mass transport limitations on the ferrocyanide oxidation, experiments were carried out at various rotation speeds of the working electrode (600, 900, and 3200 rpm). Considering the partial instability of aqueous solutions of K₄[Fe(CN)₆] and K₃[Fe(CN)₆], experiments at different rotation speeds were performed using a freshly prepared solution.

#### Algorithm

This section provides a concise description of the LF-based algorithm for determining the DRTs (DRT). This algorithm was first introduced in our prior works,^18^^,^^20^^,^^21^ with MATLAB code for its implementation available on GitHub.^43^ For comparison with the inverse-based DRT calculation method, we utilized the DRT tools—a MATLAB GUI, also accessible on GitHub.^57^

The algorithm follows these steps.

1. Sampling and data collection.
  • Sample the impedance data $Z\left(i\omega_{i}\right)$ for i=1,2,…,N, where $i\omega_{i}$ are the complex frequency points.
  • Organize the sampled data into:
    ○ Interpolation nodes: $\left\{i\omega_{i}|i=1,2,\ldots N\right\}$
    ○ Interpolation values: $\left\{Z\left(i\omega_{i}\right)|i=1,2,\ldots N\right\}$
2. Partition the data.
  • Divide the interpolation nodes and values into two disjoint sets:

$$\begin{array}{c} \left\{\omega_{1},\omega_{2},\ldots ,\omega_{N}\right\}=\underset{\text{left}\;\text{nodes}\;}{\underset{︸}{\left\{\mu_{1},\mu_{2},\ldots ,\mu_{q}\right\}}}\cup \underset{\text{right}\;\text{nodes}\;}{\underset{︸}{\left\{\lambda_{1},\lambda_{2},\ldots ,\lambda_{k}\right\}}},\text{where}\;q+k=N\text{,}\\ \left\{Z\left(i\omega_{1}\right),Z\left(i\omega_{2}\right),\ldots ,Z\left(i\omega_{N}\right)\right\}=\underset{\text{left}\;\text{values}\;}{\underset{︸}{\left\{v_{1},v_{2},\ldots ,v_{q}\right\}}}\cup \underset{\text{left}\;\text{values}}{\underset{︸}{\left\{w_{1},w_{2},\ldots ,w_{k}\right\}}} \end{array}$$

3. Construct the Loewner Matrices.
  • Use the disjoint sets to compute the following:
    ○ Loewner matrix $L\in C^{q\times k}$:
      $$L=\left[\begin{array}{cc} \begin{array}{c} \frac{v_{1}-w_{1}}{\mu_{1}-\lambda_{1}} \end{array} & \begin{array}{cc} \cdots & \frac{v_{1}-w_{k}}{\mu_{1}-\lambda_{k}} \end{array}\\ \begin{array}{c} \vdots \\ \frac{v_{q}-w_{1}}{\mu_{q}-\lambda_{1}} \end{array} & \begin{array}{cc} \ddots & \vdots \\ \cdots & \frac{v_{q}-w_{k}}{\mu_{q}-\lambda_{k}} \end{array} \end{array}\right]$$
    ○ Shifted Loewner matrix $L_{s}\in C^{q\times k}$:
      $$L_{s}=\left[\begin{array}{cc} \begin{array}{c} \frac{{\mu_{1}v}_{1}-\lambda_{1}w_{1}}{\mu_{1}-\lambda_{1}} \end{array} & \begin{array}{cc} \cdots & \frac{{\mu_{1}v}_{1}-{\lambda_{k}w}_{k}}{\mu_{1}-\lambda_{k}} \end{array}\\ \begin{array}{c} \vdots \\ \frac{{\mu_{q}v}_{q}-{\lambda_{1}w}_{1}}{\mu_{q}-\lambda_{1}} \end{array} & \begin{array}{cc} \ddots & \vdots \\ \cdots & \frac{{\mu_{q}v}_{q}-{\lambda_{k}w}_{k}}{\mu_{q}-\lambda_{k}} \end{array} \end{array}\right]$$
    ○ Column vector $V\in C^{k\times 1}$ and the row vector $W\in C^{1\times q}$:
      $$V^{T}=\left[\begin{array}{c} v_{1}\;v_{2}\;\cdots \;v_{q} \end{array}\right],W=\left[\begin{array}{c} w_{1}\;w_{2}\;\cdots \; \end{array}\text{wk}\right]$$

4. Ensure real-valued models.
  • Multiply the matrices with a block-diagonal matrix J, where:
    $$J=\text{BlockDiag}\left(J,J,\ldots ,J\right)\;with\;J=\frac{1}{2}\left[\begin{array}{cc} 1 & -i\\ 1 & i \end{array}\right]$$
  • Transform the matrices:
    $$L\leftarrow J^{*}LJ,L_{s}\leftarrow J^{*}L_{s}J,V\leftarrow J^{*}V,W\leftarrow WJ$$

5. Singular value decomposition (SVD).
  • Compute the SVD of the Loewner matrix L:
    $$L=Y\Sigma X^{*}\approx Y_{r}\Sigma_{r}X_{r}^{*}$$
    where $\Sigma_{lr},\Sigma_{rr}\in C^{r\times r},Y_{r}\in C^{q\times r},\text{and}\;X_{r}\in C^{k\times r}$.
  • The rank r is determined by the rank of the Loewner pencil $\left(L_{s},L\right)$.
  • In cases where model order reduction is required, with $r<rank\left(\left[L_{s},L\right]\right)$, it is preferable to compute the SVD of the Loewner pencil $\left(L_{s},L\right)$, given by:
    $$\left[\begin{array}{cc} L & L_{s} \end{array}\right]=Y\Sigma_{l}{\tilde{X}}^{*}\approx Y_{r}\Sigma_{lr}{\tilde{X}}_{r}^{*},\left[\begin{array}{c} L\\ L_{s} \end{array}\right]=\tilde{Y}\Sigma_{r}X^{*}\approx {\tilde{Y}}_{r}\Sigma_{rr}X_{r}^{*}$$

6. State-space model construction.
  • Compute reduced-order state-space matrices:
    $$E_{r}=-Y_{r}^{*}LX_{r},\;A_{r}=-Y_{r}^{*}L_{s}X_{r},\;B_{r}=Y_{r}^{*}V,\;C_{r}=WX_{r}$$
  • These matrices approximate the transfer function Z(iω) as:
    $$Z\left(i\omega \right)={C_{r}\left({i\omega E}_{r}-A_{r}\right)}^{-1}B_{r}$$

7. Eigenvalue decomposition (EVD).
  • Compute the eigenvalue decomposition (EVD) of the matrix $E_{r}^{-1}A_{r}$ as:
    $$E_{r}^{-1}A_{r}=V_{r}\Lambda_{r}V_{r}^{-1}\text{,}$$
    where,
    ○ $\Lambda_{r}=diag\left(\lambda_{1},\ldots ,\lambda_{r}\right)\in C^{r\times r}$ contains the poles $\lambda_{i}$,
    ○ $V_{r}=\left[v_{1},\ldots ,v_{r}\right]\in C^{r\times r}$ is the matrix of right eigenvectors,
    ○ $V_{r}^{-1}={\left[w_{1},\ldots ,w_{r}\right]}^{T}\in C^{r\times r}$ is the matrix of left eigenvectors.

8. Residue calculation.
  • For each pole $\lambda_{i}$, calculate the residue $\gamma_{i}$ of the impedance transfer function:

$$\gamma_{i}=\left[C_{r}v_{i}\right]\left[w_{i}^{T}\left(E_{r}^{-1}B_{r}\right)\right]\text{,}$$

where $v_{i}$ is the i-th right eigenvector, and $w_{i}$ is the i-th left eigenvector.

9. Determine resistances and time constants (a.k.a. DRT).
  • Using the residues and poles, calculate:
    ○ Resistances, $R_{i}$:
      $$R_{i}=-\frac{\gamma_{i}}{\lambda_{i}}$$
    ○ Time constants, $\tau_{i}$:
      $$\tau_{i}=-\frac{1}{\lambda_{i}}$$
  • The impedance transfer function, analogous to the Voigt model with multiple RC circuits in series, is expressed as:
    $$Z\left(i\omega \right)=\sum_{i=1}^{r}\frac{R_{i}\;}{1+i\omega \tau_{i}}$$
  • Plotting the resistances $\left(R_{i}\right)$ and against their corresponding time constants $\left(\tau_{i}\right)$, yields a discrete DRT, offering valuable insights into the system’s relaxation dynamics.

### Quantification and statistical analysis

A comprehensive quantification analysis was conducted, as illustrated in Figures 2C and 2D, to quantitatively assess the difference between the DRT derived from the LF-based algorithm and those obtained using the analytical approximations (aDRT) proposed in the literature to describe the FLW. The comparative accuracy is evaluated through the relative residuals, presented in Figure S1 of the supplemental information. This figure supports the analysis shown in Figures 2C and 2D by detailing the residuals between the synthetic EIS data and those generated by these two. These residuals were computed in MATLAB using the following equations:

For the real part of the impedance:

$$\Delta_{real}\left(i\omega \right)=\frac{Z_{data,real}\left(i\omega \right)-Z_{method,real}\left(i\omega \right)}{Z_{data,abs}\left(i\omega \right)}\times 100\%$$

For the imaginary part of the impedance:

$$\Delta_{imag}\left(i\omega \right)=\frac{Z_{data,imag}\left(i\omega \right)-Z_{method,imag}\left(i\omega \right)}{Z_{data,abs}\left(i\omega \right)}\times 100\%$$

Here, $Z_{data,real}$ and $Z_{data,imag}$ represent the real and imaginary components of the data, respectively, while $Z_{method,real}$ and $Z_{method,imag}$ denote the real and imaginary components of the impedance data reproduced from either the LF-based DRT or the aDRT. Additionally, $Z_{data,abs}$ represents the magnitude of the data.
