# Supplementary material for: Data-driven analysis of electrochemical impedance spectroscopy using the Loewner framework
Source: iScience. 2025 Feb 10;28(3):111987. doi: 10.1016/j.isci.2025.111987 (PMC11907484; doi:10.1016/j.isci.2025.111987)
Supplement: Document S1. Figures S1–S7, Table S1, and Data S1 and S2 [file mmc1.pdf]

iScience, Volume *28*

Supplemental information

Data-driven analysis of electrochemical impedance spectroscopy using the Loewner framework

Bansidhar Patel, Antonio Sorrentino, and Tanja Vidakovic-Koch

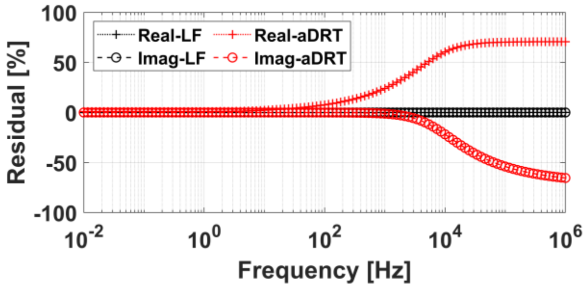

Figure S1. Relative residuals between the DRT obtained using the LF method and the analytical DRT (aDRT) from the literature [S1-2] for the FLW element, related to Figure 2

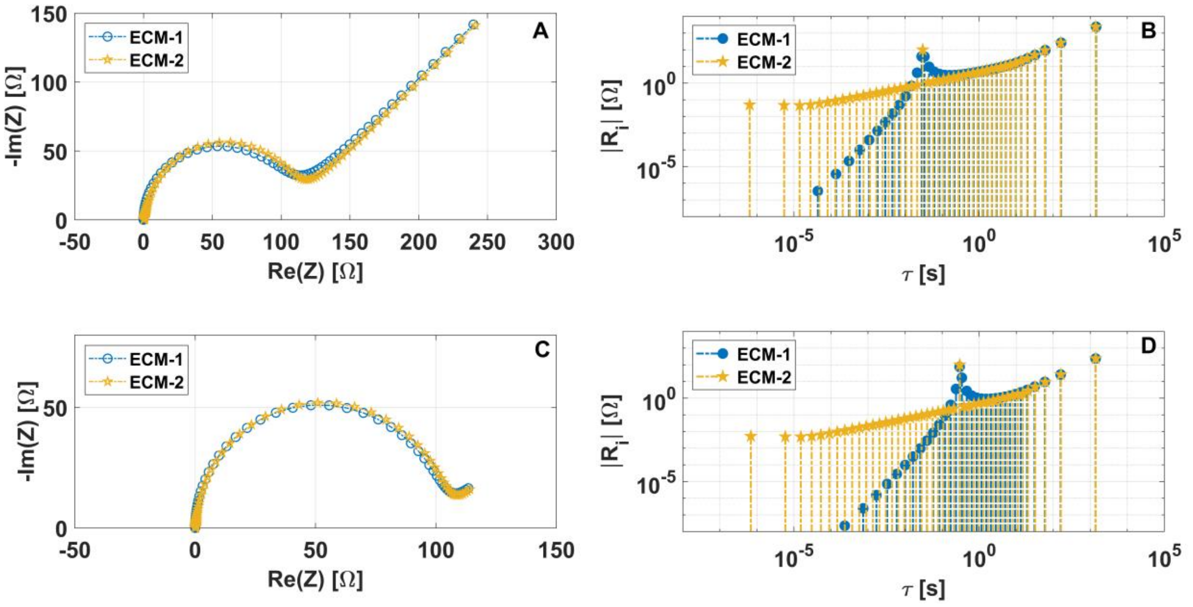

Figure S2. Analysis of simulated EIS data of the ECMs described in the paper [S3] for two sets of parameters, related to Figure 3

The ECMs are similar to ECM-1 and ECM-2 (Table 1), but with semi-infinite linear Warburg diffusion (Equation S2) instead of FLW (Equation S3) and different parameters from Orazem et al. [S3].

(A and B) Nyquist plot and corresponding DRT plot for $R_{ct} = 100, R_d = 50,$ and $C = 300\ \mu$F.

(C and D) Nyquist plot and corresponding DRT plot for $R_{ct} = 100, R_d = 5,$ and $C = 3$ mF.

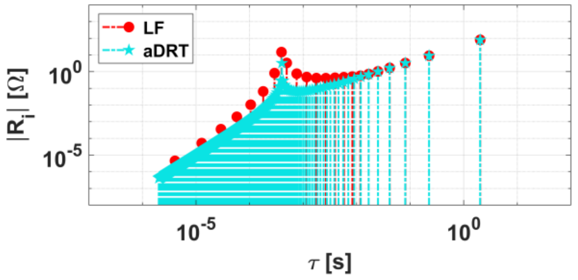

Figure S3. Comparison of the DRT obtained using the LF method with the analytical DRT (aDRT) by Montella [S2], related to Figure 3

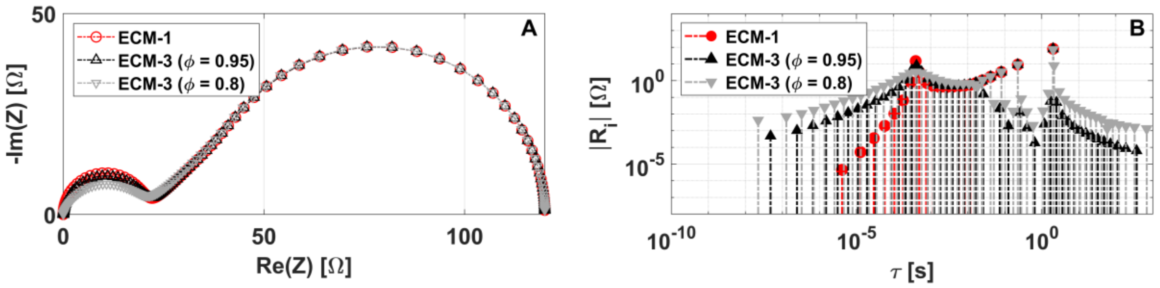

Figure S4. Analysis of simulated EIS data of the ECM-3 corresponding to Table 1 for the different values of CPE exponent (∅), related to Figure 3

(A) Nyquist plot representation.

(B) Corresponding DRT plot.

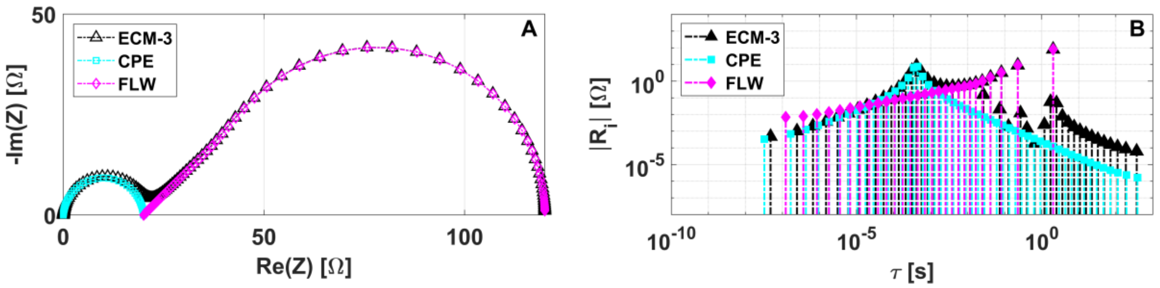

Figure S5. Analysis of simulated EIS data of the ECM-3 corresponding to Table 1, considering only R-CPE and FLW, related to Figure 3

(A) Nyquist plot representation.

(B) Corresponding DRT plot.

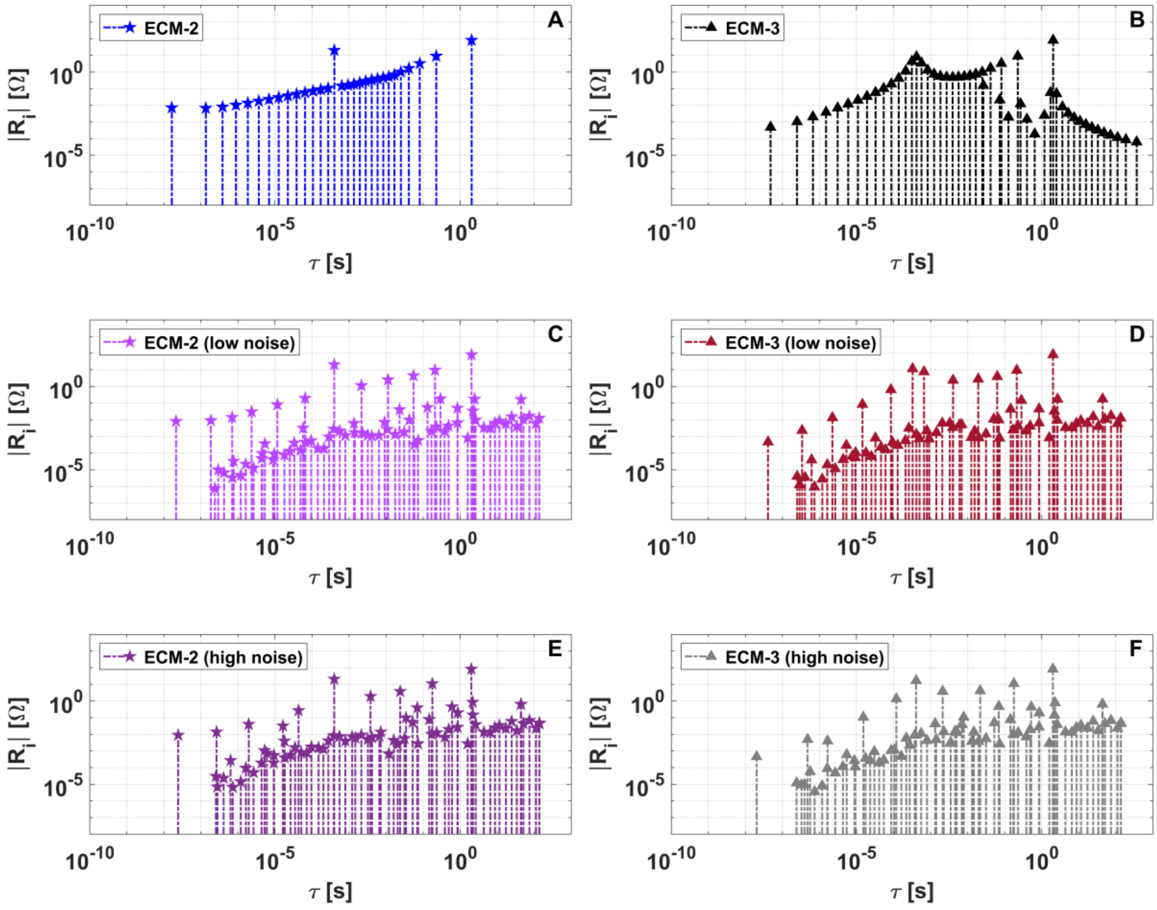

Figure S6. Analysis of simulated EIS data of the ECM-2 and ECM-3 at different noise levels, related to Figure 4

(A and B) DRT plot with no noise ($\sigma = 0$).

(C and D) DRT plot with low noise ($\sigma = 0.0005|Z|$).

(E and F) DRT plot with high noise ($\sigma = 0.002|Z|$).

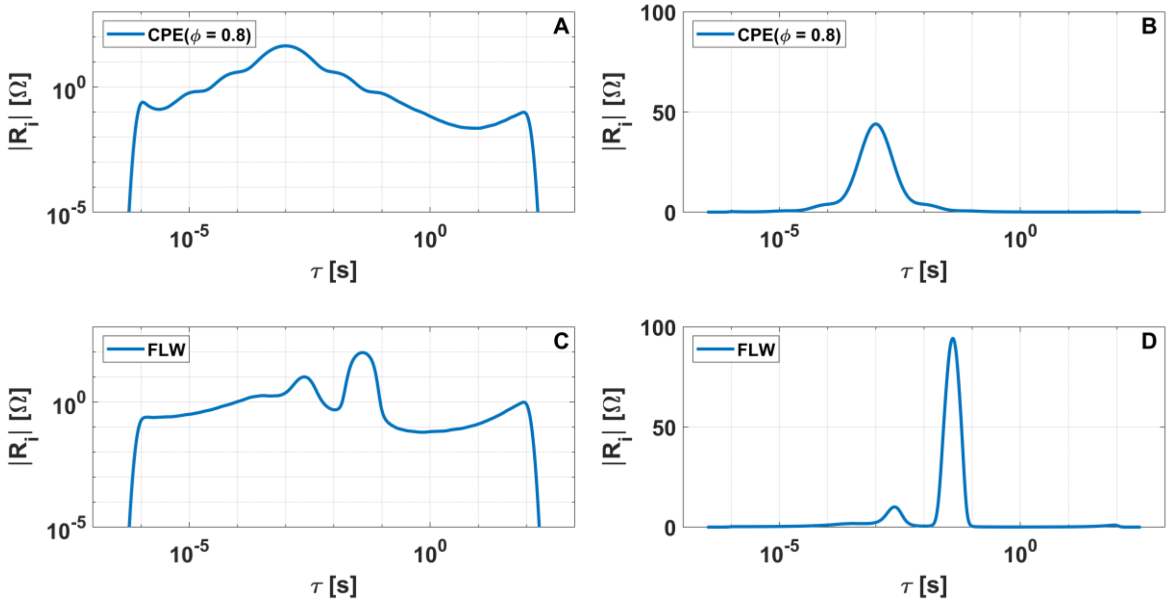

Figure S7. DRTs extracted using the algorithm presented in [S4-6] for individual elements of the ECMs presented in Table 1 (R-CPE and FLW), related to Figure 6
(A and C) DRT plots in log-logarithmic scale.
(B and D) DRT plots in semi-logarithmic scale.

Table S1. Values of the parameters used for the calculation of the EIS data for the ECMs in Table 1, related to Table 1

Parameter	Value
$\boldsymbol{C}$, F	2×10^{-5}
Ø, -	0.95
$\boldsymbol{R_0}$, Ω	0
$\boldsymbol{R_{ct}}$, Ω	20
$\boldsymbol{R_d}$, Ω	100
$\boldsymbol{\tau_d}$, s	5

Data S1. Analytical expressions of impedance and DRT of some typical equivalent circuit elements, related to Figure 2 and Table 1

The impedance of R-CPE element (parallel connection of resistance R and CPE) can be calculated based on [S7]:

$$Z_{R-CPE} = \frac{R}{1 + (j\omega)^{\emptyset} QR} \quad \text{(Equation S1)}$$

The Warburg impedance of semi-infinite linear diffusion is expressed as [S8]:

$$Z_W = \frac{R_d}{\sqrt{j\omega}} \quad \text{(Equation S2)}$$

The impedance of the finite-length Warburg (FLW) diffusion reads [S9-12]:

$$Z_W = \frac{R_d}{\sqrt{j\omega\tau_d}} tanh\left(\sqrt{j\omega\tau_d}\right) \quad \text{(Equation S3)}$$

The analytical DRT (aDRT) of the finite-length Warburg (FLW) diffusion [S1-2] in the form of the Voigt circuit model reads:

$$Z_W = \sum_{k=1}^{n} \frac{R_k}{1 + \mathrm{i}\omega\tau_k} \quad \text{(Equation S4)}$$

$$Where\ R_k = \frac{2R_d}{\pi^2 \cdot (k-0.5)^2},\ and\ \tau_k = \frac{\tau_d}{\pi^2 \cdot (k-0.5)^2}$$

The comparison between relative residuals corresponding to the DRT obtained by the LF method and the analytical DRT (aDRT) based on [S1-2] for FLW are depicted in Figure S1.

Data S2. Comparison of the DRT obtained through LF with the analytical DRT derived by Montella [S2] for ECM-1, related to Figure 3

Figure S3 compares the results obtained using LF with the analytical DRT (aDRT) recently proposed by Montella [S2] for ECM-1. A perfect qualitative agreement between the DRTs from these two methods is observed, further validating the distribution resulting from the strong coupling between kinetics and diffusion.

The aDRT of the standard Randles cell (ECM-1) according to Montella [S2] can be represented in the form of the Voigt circuit model:

$$Z(j\omega) = R_p - \sum_{k=1}^{n} R_k + \sum_{k=1}^{n} \frac{R_k}{1 + j\omega\tau_k} \qquad \text{(Equation S5)}$$

where

$$R_k = \frac{2R_d(1 + r_R q_k \cot q_k)}{q_k^2\left[1 + \frac{\cot q_k}{q_k} + (\cot q_k)^2 - r_R r_\tau q_k^2\left(1 - \frac{\cot q_k}{q_k} + (\cot q_k)^2\right)\right]}$$

and

$$\tau_k = R_k C_k$$

with

$$C_k = \frac{\tau_d}{2R_d} \cdot \frac{1 + \frac{\cot q_k}{q_k} + (\cot q_k)^2 - r_R r_\tau q_k^2\left(1 - \frac{\cot q_k}{q_k} + (\cot q_k)^2\right)}{1 + r_R q_k \cot q_k}$$

$$r_R = \frac{R_{ct}}{R_d}$$

$$r_\tau = \frac{R_d C}{\tau_d}$$

$$q_k = (2k - 1)\pi/2$$

For the derivation of this approximation, see Montella's paper [S2].

Supplemental references

[S1] Boukamp, B.A. (2017). Derivation of a Distribution Function of Relaxation Times for the (fractal) Finite Length Warburg. Electrochimica Acta *252*, 154-163. https://doi.org/10.1016/j.electacta.2017.08.154.

[S2] Montella, C. (2020). Voigt circuit representation model for electrochemical impedances under finite-length diffusion conditions. Journal of Electroanalytical Chemistry *879*, 114785. https://doi.org/10.1016/j.jelechem.2020.114785.

[S3] Orazem, M.E., and Ulgut, B. (2024). On the Proper Use of a Warburg Impedance. Journal of The Electrochemical Society *171*, 040526. https://doi.org/10.1149/1945-7111/ad3b76.

[S4] Ciucci, F., and Chen, C. (2015). Analysis of Electrochemical Impedance Spectroscopy Data Using the Distribution of Relaxation Times: A Bayesian and Hierarchical Bayesian Approach. Electrochimica Acta *167*, 439-454. https://doi.org/10.1016/j.electacta.2015.03.123.

[S5] Effat, M.B., and Ciucci, F. (2017). Bayesian and Hierarchical Bayesian Based Regularization for Deconvolving the Distribution of Relaxation Times from Electrochemical Impedance Spectroscopy Data. Electrochimica Acta *247*, 1117-1129. https://doi.org/10.1016/j.electacta.2017.07.050.

[S6] Wan, T.H., Saccoccio, M., Chen, C., and Ciucci, F. (2015). Influence of the Discretization Methods on the Distribution of Relaxation Times Deconvolution: Implementing Radial Basis Functions with DRTtools. Electrochimica Acta *184*, 483-499. https://doi.org/10.1016/j.electacta.2015.09.097.

[S7] Zoltowski, P. (1998). On the electrical capacitance of interfaces exhibiting constant phase element behaviour. Journal of Electroanalytical Chemistry *443*, 149-154. https://doi.org/10.1016/S0022-0728(97)00490-7.

[S8] Landolt, D. (2003). Corrosion et chimie de surfaces des métaux (Presses polytechniques et universitaires romandes).

[S9] Orazem, M.E., and Tribollet, B. (2008). Electrochemical Impedance Spectroscopy (John Wiley & Sons). https://doi.org/10.1002/9780470381588.

[S10] Lasia, A. (2002). Electrochemical Impedance Spectroscopy and its Applications. In Modern Aspects of Electrochemistry, B.E. Conway, J.O.M. Bockris, and R.E. White, eds. (Springer US), pp. 143-248. https://doi.org/10.1007/0-306-46916-2_2.

[S11] Fouquet, N., Doulet, C., Nouillant, C., Dauphin-Tanguy, G., and Ould-Bouamama, B. (2006). Model based PEM fuel cell state-of-health monitoring via ac impedance measurements. Journal of Power Sources *159*, 905-913. https://doi.org/10.1016/j.jpowsour.2005.11.035.

[S12] Boukamp, B.A. (2020). Distribution (function) of relaxation times, successor to complex nonlinear least squares analysis of electrochemical impedance spectroscopy? Journal of Physics: Energy *2*. https://doi.org/10.1088/2515-7655/aba9e0.
